# Strategies for Improving Quality and Safety in Global Health: Lessons From Nontechnical Skills for Surgery Implementation in Rwanda

**DOI:** 10.9745/GHSP-D-21-00042

**Published:** 2021-09-30

**Authors:** Daniel Josef Lindegger, Egide Abahuje, Kenneth Ruzindana, Elizabeth Mwachiro, Gilbert Rutayisire Karonkano, Wendy Williams, George Ntakiyiruta, Robert Riviello, Steven Yule, Simon Paterson-Brown

**Affiliations:** a University of Geneva, Geneva, Switzerland.; b Feinberg Medical School, Northwestern University, Chicago, IL, USA.; c University of Rwanda College of Medicine and Health Sciences, Kigali, Rwanda.; d Tenwek Mission Hospital, Nairobi, Kenya.; e Rwanda Military Hospital, Kigali, Rwanda.; f Brigham and Women’s Hospital, Boston, MA, USA.; g Harvard Medical School, Boston, MA, USA.; h Ejo Heza Surgical Center, Kigali, Rwanda.; i NHS Lothian University Hospitals Division, Edinburgh, Scotland.

## Abstract

The Non-Technical Skills for Surgeons (NOTSS) framework is a taxonomy of cognitive and social skills that foster expertise and medical knowledge in the operating room. This framework can be used as a method to improve the quality of surgical care in global efforts to improve access to affordable surgery.

## Key Messages

- Efforts to increase access to surgical care will achieve improved health outcomes only if those efforts are intertwined with efforts to increase surgical safety and quality.
- The World Health Organization Surgical Safety Checklist and Non-Technical Skills for Surgeons (NOTSS) framework are 2 tools to increase surgery quality and safety.
- The NOTSS for variable resource contexts is a new 1-day educational course developed in Rwanda that integrates contextually appropriate behaviors and values and can be implemented with low costs in any health care context.
- Program managers should provide context-specific NOTSS training for surgeons and operating theater staff.
- Policy makers should implement NOTSS framework into health care policy focusing on modern virtual teaching methods.
- Surgeons and operating theater staff should be familiar with the NOTSS framework and regularly update their knowledge through didactic courses, simulation, and online trainings.

Résumé en français à la fin de l'article.

## INTRODUCTION

In 2015 the Lancet Commission on Global Surgery published its report “Global Surgery 2030: evidence and solutions for achieving health, welfare, and economic development,”^1^ helping to galvanize a global movement to increase access to safe, timely, and affordable surgical and anesthesia care with an emphasis on equity. A goal of the movement is to enable the benefits of these efforts to be reaped most by impoverished and marginalized populations. The authors laid out 5 key messages, including the great number of operations required annually (approximately 143 million), especially among the poorest third of the world’s population, which receives only 6% of the operations. The commission called on nations to track and report on 6 metrics related to surgical care. Two of these metrics—surgeon, anesthetist, and obstetric (SAO) density (the number of specialist surgical, anesthetic, and obstetric providers per 100,000 population) and surgical volume (number of operations performed in operating rooms annually per 100,000 population)—are measurements of surgical delivery. To achieve the proposed targets of 20 SAOs and 5,000 operations/100,000, the surgical workforce and the capacity to perform surgery must be greatly expanded.

Such efforts to grow the surgical workforce are currently being undertaken by regional professional colleges such as the College of Surgeons of East, Central, and Southern Africa,^2^ the Pan-African Academy of Christian Surgeons,^3^ the West African College of Surgeons,^4^ and country-specific health professions training institutions. These efforts, when tied to initiatives to strengthen essential surgical care at district hospitals, hold promise for substantially increasing access to surgical care for destitute sick and injured populations and individuals served at these facilities. The current global COVID-19 pandemic has affected access to surgical care because most health resources have been mobilized toward the prevention and management of patients with COVID-19. A total of 28,404,603 surgical procedures are estimated to have been canceled globally due to COVID-19. In sub-Saharan Africa, 520,458 surgical procedures were estimated to have been canceled due to COVID-19.^5^

Efforts to increase surgical volume will achieve greater measures of health and economic wellness but only if surgery is of high quality. In contrast, expanded access to unsafe, poor-quality surgery will increase complications and surgical deaths, in essence, trading the disease burden of untreated surgical conditions for an epidemic of iatrogenic perioperative complications. This problem may be developing even now. For example, countries throughout Africa have invested in increasing access to cesarean delivery, which is needed to improve maternal and neonatal safety by treating obstructed labor and other complications of delivery. Proper and timely treatment of obstructed labor will decrease maternal and neonatal death rates and also eliminate obstetric fistula. While obstetric fistula due to untreated obstructed labor is decreasing, the incidence of iatrogenic vesicovaginal fistula and surgical site infections due to poorly performed cesarean delivery is on the rise.^6^ Hence, it is imperative that efforts to increase safety and quality in surgery be intertwined with efforts to increase access.

Efforts to increase surgical volume will achieve greater measures of health and economic wellness, but only if surgery is of high quality.

The World Health Organization Safe Surgery Checklist was developed to increase the safety of operating teams by encouraging communication and establishing shared mental models for teams performing surgical operations. Similarly, the Non-Technical Skills for Surgeons (NOTSS) framework was developed to improve intraoperative patient safety. NOTSS are defined as social (leadership, communication, and teamwork) and cognitive (situational awareness and decision making) skills that underpin medical knowledge, technical skills, and appropriate use of resources.^7^

The NOTSS framework has 4 categories, and each category has 3 elements (Figure). The framework provides scaffolding for teaching and assessing that has been demonstrated to be useful across multiple contexts that vary richly in language, culture, resources, level of care in the health system, and surgical epidemiology.^8^^–^^11^ Finally, by emphasizing team training, since surgical care is delivered by multidisciplinary groups of health care specialists, updated versions of NOTSS serve as a model of team care that will increase safety and quality throughout health systems. This article describes how lessons learned from NOTSS implementation in Rwanda can be used to improve quality and safety in global health programs. Based on our experience in developing and implementing a NOTSS training program in Rwanda, we describe strategies that can be used to improve the quality of surgical care in efforts to improve access to timely and affordable surgery.

**FIGURE:**
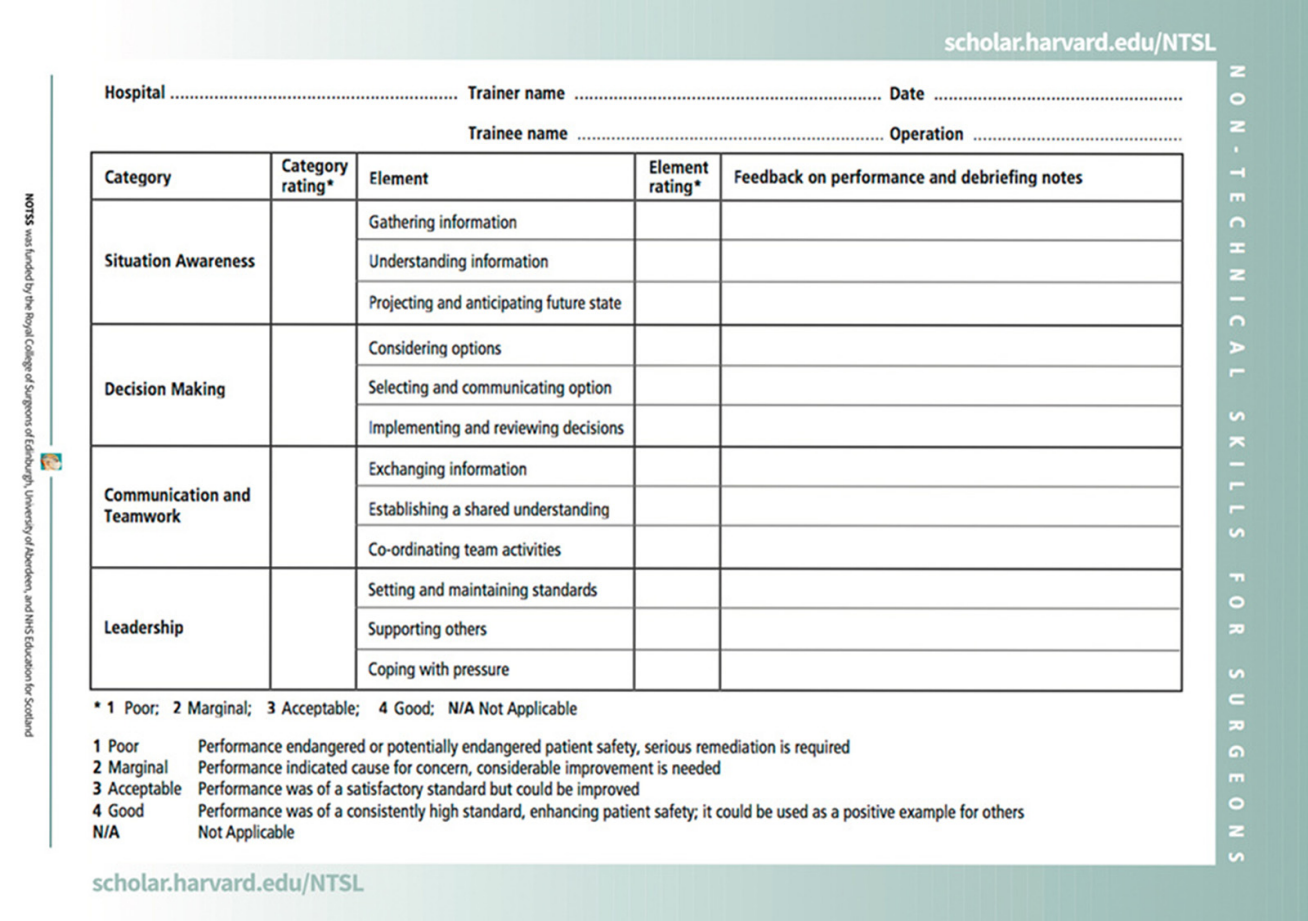
Nontechnical Skills for Surgeons Rating Tool

## NOTSS AND PATIENT SAFETY

*Nontechnical skills* (NTS) is a behavioral construct that originated in the aviation industry. An analysis of root causes of airplane crashes revealed that these events were not only due to lack of technology and technical skills but also a result of ineffective human factors among crew members. Crew resource management was subsequently developed as a way of reducing airplane disasters.^12^ Similar to the airline industry, 80% of errors in anesthesia^13^ and more than 50% of intraoperative surgical errors are due to ineffective communication between members of the surgical team.^14^ Crew resource management transitioned to the health care industry through various NTS courses that were developed by different disciplines. In this regard, Anesthetists’ Non-Technical Skills, Scrub Practitioners’ List of Intra-operative Non-Technical Skills, and NOTSS were developed and largely taught in respective disciplines. In this article, we use the NOTSS framework as an example to describe how safety and quality of care can be improved as global access to care is increased.

Improvement of NOTSS can be achieved by appropriate training^15^^,^^16^ that can be integrated into undergraduate, postgraduate, and continuous health professions education curricula.^16^ NOTSS for variable-resource contexts (NOTSS-VRC) is a novel educational curriculum for NTS for surgery that was developed in Rwanda by integrating contextually appropriate values and implemented as a 1-day course for surgical teams (general practitioners/surgeons, perioperative nurses, and anesthetists).

Before implementing the NOTSS course in Rwanda, Scott et al.^9^ conducted a systematic review to understand the contextual challenges to patient safety in low- and middle-income countries (LMICs). They identified that overburdened health care systems, lack of provider empowerment, and deficiencies in provider training compromise safe patient care in LMICs. Furthermore, a needs assessment to understand the critical NTS for surgeons needed for high performance in LMICs was conducted. This assessment identified that the categories of high NOTSS performance required in LMICs were identical to those in high-income countries, but the examples of specific behaviors indicative of those skills were different.^11^ Based on these findings, Lin et al.^8^ developed a NOTSS-VRC consisting of teaching videos, didactic lectures for a 1-day course, and a handbook for instructors. The curriculum can be implemented without significant financial cost in a resource-constrained country as an educational or quality-improvement strategy.

The categories of high NOTSS performance required in LMICs are identical to those in high-income countries, but the specific behaviors indicative of those skills are different.

During course implementation, instructors collected feedback from participants. Based on this information, the course was revised and expanded to include other members of surgical teams (obstetricians, gynecologists, anesthetists, and perioperative nurses).

To accommodate all members of the surgical team; the terminology and examples of skills indicative of effective and ineffective behaviors were revised and they integrate all specialties. In addition, local instructors were trained and supported to teach NOTSS to surgical care providers from Rwandan district hospitals.

Although there are no patient-related data from Rwanda, a report by Scott et al.^11^ showed that understanding of NOTSS among residents improved significantly after taking the course, which is a strong argument for its usefulness in LMICs. Evaluation of the NOTSS-VRC course showed that participants’ knowledge of NTS improved after the course. In addition, the training was perceived as “enjoyable, practical and informative” by the course participants.^8^ Furthermore, Abahuje et al.^17^ found that after completing a NOTSS program, participants were able to translate NTS in a clinical environment, and they perceived that the NOTSS program empowered them to speak up and helped them to improve team dynamics and to provide safe patient care. Similarly, Mossenson et al.^18^ evaluated the impact of the Vitals Anesthesia Simulation Training (VAST) course in Rwanda. The VAST course is a 3-day simulation-based program that teaches core clinical skills and NTS required by anesthesia providers in limited-resource settings. This evaluation showed that participants acquired and retained knowledge up to 3 months after completing the VAST course. Participants reported that after taking the course, they were empowered to advocate for better patient care and system improvement.

Gordon et al.^19^ carried out a systematic review to analyze the effect of different NOTSS training interventions on patient safety. Over half of the 22 analyzed studies described interventions delivered to multidisciplinary teams. The teaching methods included simulation and role-playing, observation, didactic teaching, and games. The authors acknowledged that the analyzed studies had significant heterogeneity, which hindered drawing conclusions on the effectiveness of the interventions. However, most studies reported positive outcomes of the teaching interventions, which suggests they have educational utility. The content themes of the studies analyzed were communication, error, systems, team working, leadership, and situational awareness.

We argue that the NOTSS training program may improve safety while global access to surgical care is being expanded. Based on our experience of developing and implementing the NOTSS-VRC program in Rwanda, we recommend teaching NOTSS at the hospital level because that is where health care providers, who were trained in different centers, meet and work. The course can be easily contextualized to address the challenges of the course participants, and the opportunity exists to assess the patient-related impact of the course. Teaching NOTSS should go beyond knowledge transfer in the classroom, simulation center, or online environment to support and provide mentorship to course participants as they implement NTS in the clinical environment. Effective NTS training needs to recognize the barriers and facilitators of skills implementation in clinical practice and help trainees to use facilitators to overcome the challenges. After taking the NTS course in Rwanda, health care providers identified an unpredictable working environment, work overload, hierarchy, and lack of interdisciplinary communication as barriers to behavior change. Most of these barriers can be addressed by the effective implementation of a NOTSS teaching program.^17^

The NOTSS training program may improve safety while global access to surgical care is being expanded.

## STRATEGY FOR TEACHING NOTSS

During course preparation, instructors have to understand factors that may affect the ability of translating NOTSS into clinical practice and tailor the course in a way that enables learners to acquire skills necessary to overcome these barriers. For example, they can learn how to communicate with their superiors and improve communication with people from other disciplines, how to prioritize work when they are overloaded, and how to optimize patient care in a variable-resource setting.

Following the course, trainees should develop an action plan or quality improvement projects. Action plans ensure that learners continue to use their new skills and teach them to colleagues, thereby improving the quality of services at their facilities. Through mentorship, the instructors need to continuously mentor trainees and support them as they implement their quality improvement projects. Mentorship can be delivered through virtual online group discussions as well as onsite visits by the instructors. Follow-up and supportive supervision are key to helping participants apply new skills in real life. The instructors and the participants would have to agree on the frequency of the mentorship meetings, depending on the progress of the participants on their projects and the amount of support they need from the instructors. Using performance standards (harmonized and standardized with training materials) within a post-training follow-up approach or supportive supervision system can also support performance improvement. Following the increased popularity of virtual teaching sessions in the COVID-19 era, refresher courses can be easily organized and thus allow long-term assessment through questionnaires, which will provide evidence on how the skills taught in the course are implemented in the workplace environment and retained by the trainee.

To make an impact on the local surgical training landscape, integration of the NOTSS-VRC course into the university curriculum is desired. Involvement of other partners such as the Ministry of Health, Professional Societies (e.g., surgical, anesthesia, and nurse societies) may allow the implementation of the framework on a national scale and allow for continuous iterative development of the taxonomy to the local needs.^8^

## SCALING UP NTS

Expansion of NOTSS to new geographical areas should be preceded by a contextual and needs assessment to understand the factors that may have an impact on the successful implementation of the course. Findings from this assessment inform the design and delivery of the course. Although Rwanda has many similarities with other LMICs, the steps that we used while developing and implementing a NOTSS training program in Rwanda may not transfer exactly to other LMICs. However, similar processes may be used to identify context-specific factors to facilitate the development and implementation of a NOTSS training program in any country. Future steps include expanding NOTSS to reach the College of Surgeons of East, Central, and Southern Africa, the Pan African Academy for Christian Surgeons, and the West African College of Surgeons surgical training programs. The COVID-19 pandemic has challenged educators, and technology was leveraged to meet educational needs. From this experience, we anticipate being able to produce online and blended NOTSS courses and reach people from different geographical areas.

During the COVID-19 pandemic, technology was leveraged to meet educational needs, and these lessons may help in offering NOTSS courses to people in different geographical areas.

## STRATEGIES TO ASSESS THE OUTCOME OF NOTSS EDUCATION PROGRAMS

Similar to other educational programs, NOTSS can be assessed through different levels using the Kirkpatrick model of educational program appraisal.^20^ The first level consists of an assessment of course participants’ reactions to the course. The second level assesses acquisition of knowledge, skills, and attitudes and involves pre- and posttest evaluations.^20^ The third level of this model assesses the behavior change or transfer of learning to the clinical environment. This level can be assessed by direct observation in the clinical environment or by asking participants if they implemented the NTS in a clinical environment. The fourth level assesses the impact of the course on organizational practice and patient outcomes.^20^ The majority of the educational program assessments focus on the first 2 levels. Assessment of the higher levels of the Kirkpatrick model is complex because multiple confounding factors may affect the outcomes of interest.^20^

We acknowledge the paucity of data on the impact of NOTSS training programs on behavior change and patient outcomes. Our future NOTSS training programs will be delivered at the institution; we will assess NTS behaviors of participants before and after the course, and collect patient-related metrics before and after implementing the program. This will enable gaining insight into the impact of the NOTSS training program on patient outcomes.

## CONCLUSION

The current paradigm of global surgery emphasizes an equitable increase in access to surgical delivery to counteract the global imbalance that disadvantages the world’s impoverished and marginalized populations. Surgeon density and surgical volume are important outcome measurements to assess institutions’ efforts to increase surgical care delivery. However, high-quality surgical care is equally important to prevent complications, surgical death, and reputation loss. Safe surgery will ensure that the disease burden of untreated surgical conditions is not exchanged for an epidemic of iatrogenic perioperative complications.

The World Health Organization Safe Surgery Checklist and the NOTSS framework are tools to increase quality and safety in the operating room by improving team performance through encouraging communication and by providing a taxonomy of cognitive and social skills. These tools can be used by global surgery interventions to improve access to safe and affordable surgical care.

NOTSS-VRC developed in Rwanda in 2013 is an educational curriculum implemented as a 1-day course for surgical teams practicing in VRCs that provides a practical guide for NTS. The framework encourages trainees to establish an action plan or a quality improvement project to teach acquired skills to colleagues, thus allowing scale-up of the training program. The outcome of the education program can be assessed using the 4 levels of the Kirkpatrick model. Lessons learned from the implementation in Rwanda include the importance of context-specific adaptation of the framework and inclusion into local institutions to increase long-term success of the program.

The efforts to improve global surgical care have been compromised by the COVID-19 pandemic, which has led to a shift of health care resources towards prevention and management at the cost of surgical care. Similarly, educators have been challenged because didactic or simulation-based training programs were compromised. However, the crisis has paved the way toward online and blended NOTSS courses that are forward-looking teaching modalities to reach people from different geographical areas.

## Translation

**En français**

**Stratégies d'amélioration de la qualité et de la sécurité en santé mondiale: leçons tirées dans le développement et l’exécution d’ un programme de formation des compétences non techniques de la chirurgie au Rwanda**

**Message clé pour TOC :** Les compétences non techniques pour les chirurgiens (NOTSS) est une taxonomie des compétences cognitives et sociales qui complètent l'expertise et les connaissances médicales dans la salle d'opération. NOTSS peut être utilisé comme une méthode pour améliorer la qualité des soins chirurgicaux dans les efforts mondiaux visant à améliorer l'accès à une chirurgie abordable.

**Message clé :**

- Les efforts visant à accroître l'accès aux soins chirurgicaux ne permettront d'améliorer les résultats de santé que si ces efforts sont étroitement liés aux efforts visant à accroître la sécurité et la qualité de la chirurgie.
- La liste de contrôle de la sécurité chirurgicale de l'Organisation mondiale de la santé et le cadre des compétences non techniques pour les chirurgiens (NOTSS) sont deux outils qui peuvent améliorer la qualité et la sécurité de la chirurgie.
- Le cadre NOTSS pour les contextes de ressources variables est un nouveau cours éducatif d'une seule journée développé au Rwanda qui intègre des comportements et des valeurs contextuellement appropriés et peut être mis en œuvre à faible coût dans n'importe quel contexte de soins de santé.
- Les directeurs de programme doivent fournir une formation sur compétences non techniques spécifique au contexte pour les chirurgiens et le personnel du bloc opératoire.
- Les décideurs devraient mettre en œuvre le programme to compétence non-techniques dans la politique de soins de santé en se concentrant sur les méthodes modernes d'enseignement virtuel.
- Les chirurgiens et le personnel du bloc opératoire doivent se familiariser avec les compétences non-techniques et mettre régulièrement à jour leurs connaissances par le biais de cours didactiques, de simulations et de formations virtuelles.

**Peer Reviewed**

**First published online:** August 9, 2021.
